# Electroencephalography differences in children with and without bilateral cerebral palsy during unimanual and bimanual drumming tasks

**DOI:** 10.3389/fnhum.2025.1694812

**Published:** 2026-01-05

**Authors:** Arjun Mathur, Thomas C. Bulea, Julia Kline, Diane L. Damiano

**Affiliations:** Neurorehabilitation and Biomechanics Section, Rehabilitation Medicine Department, Clinical Center, National Institutes of Health, Bethesda, MD, United States

**Keywords:** brain imaging, pediatric, upper limb, bimanual coordination, event-related desynchronization

## Abstract

**Background:**

Multiple studies have examined bimanual coordination in children with unilateral cerebral palsy (CP) with few in bilateral CP and none utilized electroencephalography (EEG). This study investigates brain activation underlying bimanual performance in individuals with bilateral CP and typical development (TD).

**Methods:**

Twenty-six participants (13 CP; 13 TD) completed the Box and Block Test (BBT) and visually cued drumming tasks with each hand (unimanual) and then with both hands synchronously and asynchronously (bimanual). EEG and motion data were recorded during drumming tasks.

**Results:**

Children with CP demonstrated bilateral impairments in drumming cadence and BBT, increased alpha and beta and decreased gamma EEG band activation in mainly non-dominant brain regions compared to TD. Bimanual tasks tended to show decreased performance and greater alpha and beta band activation than unimanual tasks for both groups. EEG activity and BBT correlations were positive in TD, but negative in CP.

**Discussion:**

This study showed that children with CP had worse motor performance bilaterally and EEG activation differences from TD similar to previous unimanual findings in bilateral CP, however, a more complex bimanual task may have uncovered greater differences.

**Conclusion:**

Evidence of bimanual deficits and EEG differences reinforces the need for greater research and clinical attention on upper limb function in bilateral CP.

## 1 Introduction

Cerebral palsy (CP) is a group of common neurodevelopmental disorders caused by injuries or insults to the brain during early development that primarily affect motor function (Gulati and Sondhi, 2018). Spastic CP, the most common sub-type, can be further differentiated as unilateral or bilateral depending on whether motor limitations are on one or both sides of the body, respectively. Until recently, most studies on upper limb function have primarily focused on children with unilateral CP, even though children with bilateral CP also experience upper limb deficits. We previously examined motor performance and the associated brain activation in children with bilateral CP and typical development (TD) during unimanual reaching, cube transfer across a midline, and button press in response to a light cue (Phillips et al., 2023; Hinchberger et al., 2023; Lee et al., 2025). As a group and across tasks, participants with bilateral CP had poorer unimanual motor performance when using both the dominant and non-dominant hand compared to the group with TD, as well as differences in brain activation patterns that were correlated with the degree of motor impairment. The goal of this study is to evaluate bimanual compared to unimanual motor performance and the associated brain processes in cohorts with bilateral CP and TD.

Bimanual coordination is the ability to use both hands in a competent manner to complete a task and involves integrating the movement of both hands. Bimanual coordination skills such as buttoning a shirt or opening a jar are critically important in everyday life. Woytowicz et al. (2016) differentiated bimanual coordination patterns as being symmetric, that is, both hands performing the same activity, or asymmetric, i.e., each hand performing a different activity. Symmetrical coordination was further sub-grouped as hands moving in-phase, anti-phase, or in complex spatial–temporal patterns, for example, pushing a cart with both hands, reciprocal arm movement on an elliptical device, or playing a piano concerto, respectively. Asymmetric bimanual activities involve different movements for each hand and can be complementary, that is, performing the same task with each hand having a different role, or independent, that is, performing different tasks with each hand. In the current study, the drumming task consisted of two unimanual (dominant and non-dominant hand) tasks and symmetric in-phase (synchronous) and anti-phase (asynchronous) tasks.

Bimanual coordination deficits are well-documented especially in unilateral CP, and strong evidence supports the efficacy of intensive unimanual (Hoare et al., 2019) and bimanual training (Gordon et al., 2007). Hung et al. (2004) compared bimanual coordination in children with *unilateral* CP and TD during a task in which participants opened a drawer with one hand and activated a light switch with the other, an independent asymmetric bimanual task. Children performed this task at a self-paced speed and an as-fast-as-possible speed, with each hand performing both roles. Children with CP were slower and less coordinated than the children with TD; however, their performance was better for the fast speed. Inter-hand timing was also better in children with unilateral CP when the more affected hand opened the drawer, because the less affected hand was able to accelerate the button press movement to better coincide with (compensate for) the drawer opening by the slower hand.

Herard et al. (2025) utilized the identical experimental paradigm to compare bimanual performance in children with bilateral CP and TD. Children with bilateral CP performed the task more slowly and sequentially than children with TD. Timing of the two hands was better when the less affected hand opened the drawer, opposite to what was observed in unilateral CP. The authors noted that the less affected hand in these children was more impaired than in unilateral CP and could not compensate (speed up) for the slower non-dominant hand as in unilateral CP.

Brain imaging has been utilized to evaluate the amount, location, or both of brain activity during bimanual coordination tasks (Bleyenheuft et al., 2015a) as well as the effects of intervention (Araneda et al., 2021) largely in unilateral CP, using either functional magnetic resonance imaging (fMRI) (Bleyenheuft et al., 2015b; Araneda et al., 2021) or functional near-infrared spectroscopy (fNIRS) (de Campos et al. 2020). For example, de Campos et al. (2020) utilized fNIRS to evaluate brain activation in children with unilateral CP compared to children with TD during bimanual in-phase and anti-phase symmetric squeezing tasks and a bimanual asymmetric pouring task, in which one hand held an empty glass while the other filled it with water from a second glass, with each hand performing both roles. Compared to the children with TD, those with unilateral CP had more diffuse and higher levels of overall brain activation in all tasks. Bilateral activation was greater in the sensorimotor areas of children with CP during anti-phase squeezing compared to children with TD, as was activation in the lesioned hemisphere during symmetrical squeezing and when either hand was pouring. Notably, more lateralization to the dominant hemisphere during asymmetrical squeezing was associated with better function.

In a related investigation, Sukal-Moulton et al. (2020) compared brain activation patterns using fNIRS during a non-dominant hand squeezing task in children with bilateral and unilateral CP and TD. The children with unilateral CP showed significantly higher activity compared to those with bilateral CP, and both groups with CP had higher activation compared to children with TD. A trend toward more bilateral or ipsilateral activation was seen in unilateral CP compared to more contralateral activation in the groups with bilateral CP and TD. Bilateral CP was, however, less strongly contralateral on average than TD. This concurs with the findings from Phillips et al. (2023) that children with bilateral CP are not as lateralized during unimanual tasks as children with TD when using the more affected side. Hand preference is a normal characteristic. In bilateral CP, one hand also tends to have better function although the differences between hands may be more subtle compared to those with unilateral CP and in some cases compared to TD.

To our knowledge, this is the first electroencephalography (EEG) study in bilateral CP that incorporates brain imaging with temporal–spatial data during an upper limb bimanual coordination task, specifically drumming. Four conditions were performed, hitting the drum with the dominant hand, the non-dominant hand, both hands together, and alternating hands performing in-phase and anti-phase symmetrical motions. This task was designed to be engaging for young participants, yet simple for many with bilateral CP to be able to perform even with their more affected hand, while enabling us to evaluate behavior and brain activation when using both hands together in two different ways, compared to when using either their dominant or non-dominant hand. Given the asymmetry across sides in unimanual motor speed and performance (Phillips et al., 2023), we anticipated this would also negatively affect bimanual performance. Since children with CP may exhibit mirror movements indicating some degree of control of both hands when trying to move only one (Kuo et al., 2018), we anticipated that synchronous tasks would be easier than asynchronous ones when trying to use both hands. Event-related spectral perturbations (ERSPs) in three frequency bands were analyzed: alpha (6 Hz to 12 Hz), beta (13 Hz to 30 Hz) and gamma (31 Hz to 50 Hz). Of note, we expanded the lower range of the alpha band based on our earlier findings that some children with CP may have lower peak alpha band activity (George et al., 2021). The alpha and beta bands typically experience a drop in power just before and during real or imagined movements indicating that the firing of the neurons is less coherent than during baseline, referred to as event-related desynchronization or ERD. The alpha and beta bands are both integrally involved in movement planning and execution, although their roles have been shown to vary (Borra et al., 2023; Mustile et al., 2024; Zaepffel et al., 2013). Specifically, the alpha band is thought to be more involved in disengaging task-irrelevant ipsilateral cortical regions while the beta-band is directly involved in disinhibition of contralateral regions related to movement preparation and in the computations of movement parameters for execution, as shown by its correlations with EMG activity and greater somatotopic organization (Brinkman et al., 2014). Greater gamma band event-related synchronization (ERS) is believed to be related to corticospinal tract efficiency and greater cortico-muscular coherence. It is typically observed in response to a visual cue and often continues during more complex tasks, with greater ERS associated with greater beta band ERD (de Lange et al., 2008; Mehrkanoon, et al., 2014). Like our EEG studies of unimanual tasks in bilateral CP, we expected higher magnitude alpha and beta band ERD but lower gamma ERS in children with bilateral CP compared to the children with TD across all tasks. We hypothesized that timing relative to the cue and beat maintenance (i.e., cadence) will be poorer during the bimanual compared to the unimanual drumming conditions in the group with CP with asynchronous and non-dominant hand tasks showing relatively poorer performance compared to synchronous and dominant hand tasks, respectively. We expected to find similar patterns of differences across conditions with greater ERD in alpha and beta and less ERS in the gamma band in conditions with poorer behavioral outcomes.

## 2 Methods

### 2.1 Participants

Sixteen typically developing (TD) children and 16 children with bilateral cerebral palsy (CP) were recruited. Three participants with CP and three participants with TD were excluded from EEG analysis due to either poor EEG quality or inability to perform the tasks as instructed. The final EEG dataset included 13 participants with CP (age 13.6 ± 3.0 years) and 13 participants with TD (age 14.5 ± 2.1 years). All participants were 6–19 years of age at the time of enrollment with no significant age difference between groups (*p* = 0.35). See Table 1 for participant details. Inclusion criteria were age 5 years or older, no surgery in the previous 6 months on upper or lower extremities, no botulinum toxin injections within 4 months, either a healthy volunteer or with a diagnosis of bilateral CP, and able to understand and follow simple directions that included how to perform a repetitive task and when to start and stop the task. Exclusion criteria included: any neurological, musculoskeletal, or cardiorespiratory injury, health condition, or diagnosis other than CP that would affect the ability to mentally concentrate or move a body part repetitively for short periods of time, uncontrolled seizures, or concurrent use of medicines for muscle tone. The study was approved by the National Institutes of Health Institutional Review Board with informed consent and assent obtained from the parent and participant, respectively.

**Table 1:** Individual participant characteristics summarized by group, cerebral palsy (CP) or typically developing (TD).

Identifier	Age (years)	Handed	Sex	GMFCS	MACS	Gestational age (weeks)	Etiology
CP1	14.4	Right	Male	I	II	38	Dyskinetic CP of unknown etiology
CP2	16.0	Left	Female	I	I	31 (twin)	PVL
CP3	17.2	Right	Female	III	I	28	PVL
CP4	14.3	Left	Male	II	I	29 (twin)	PVL suspected (no MRI)
CP5	6.5	Right	Male	II	I	37	Spastic CP (abnormal MRI; unknown cause)
CP6	14.4	Left	Female	I	I	29 (twin)	IVH
CP7	14.4	Right	Female	II	II	39–40	Dystonic CP with R hand congenital deformity
CP8	12.7	Right	Female	III	II	29	PVL
CP9	14.0	Left	Male	II	I	39	Spastic CP (normal MRI)
CP10	17.0	Left	Female	I	II	41	Dyskinetic CP
CP11	9.3	Right	Male	II	I	30	PVL
CP12	13.3	Left	Female	III	II	34–35	PVL
CP13	13.0	Right	Male	III	II	31	PVL
CP (n = 13)	13.6 ± 3.0	7 right/6 left	6 males/7 females	4I/5II/4III	7I/7II/2III		
TD (n = 13)	14.5 ± 2.1	11 right/2 left	7 males/6 females				

GMFCS, gross motor function classification system; MACS, Manual Abilities Classification System; PVL, periventricular leukomalacia; IVH, intraventricular hemorrhage; MRI, magnetic resonance imaging.

### 2.2 Experimental design

#### 2.2.1 Box and block test (BBT)

Before EEG data collection, participants performed the box and block test (BBT) (Platz et al., 2005). Each hand was tested individually. A compartment that initially contained 150 blocks was placed on the side being tested, and an initially empty compartment was positioned in front of the opposite hand. The participant laterally transferred one block at a time to the empty compartment as fast as they could for 60 s with the total number of blocks transferred recorded.

#### 2.2.2 Experimental task

**Figure 1:**
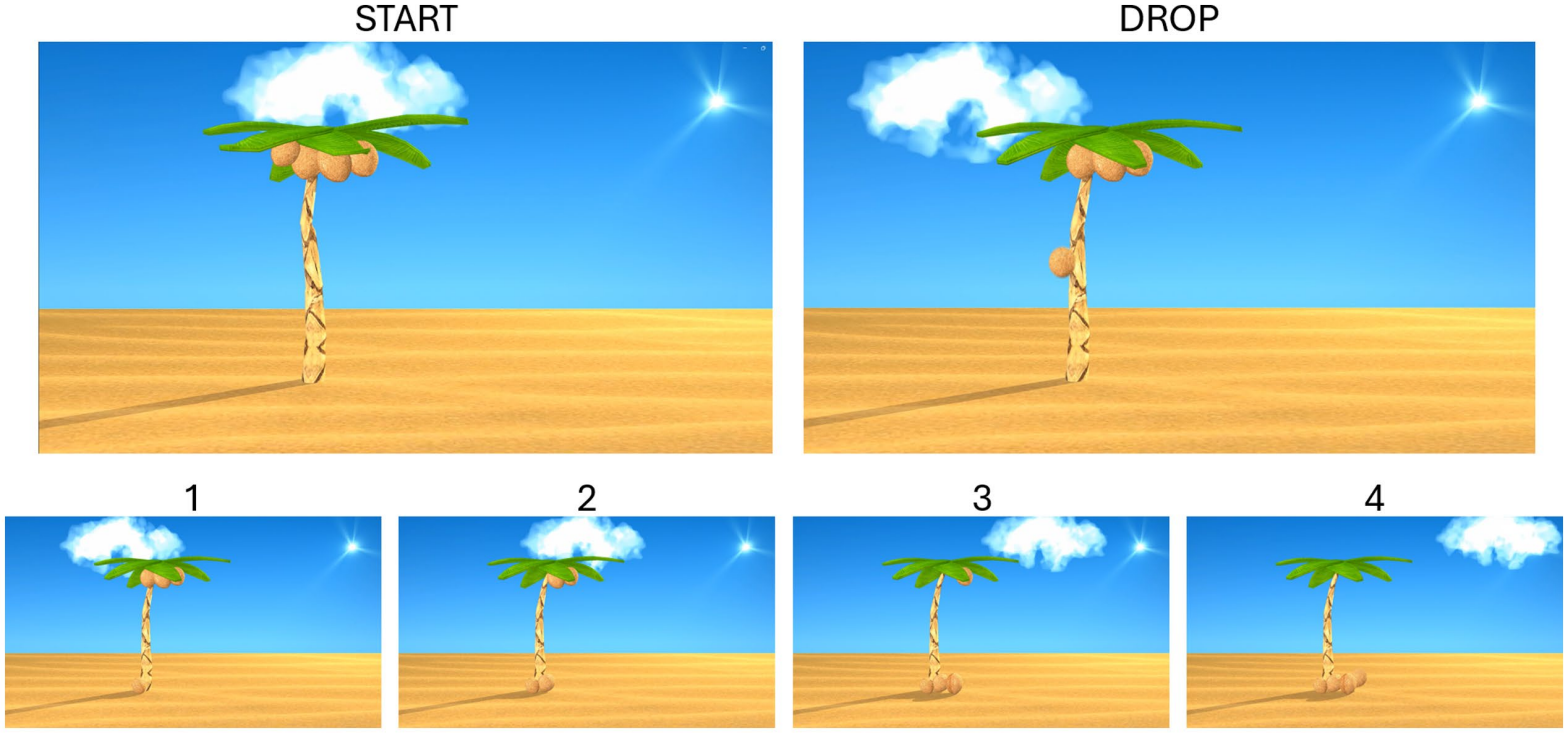
Screenshots of the video monitor to scale showing the four coconuts in the tree at the start of the trial, during a drop, and after each one dropped (bottom row).

The participants were seated comfortably facing a table with two custom-built drums. The drums were constructed using circular cardboard boxes with disks placed on top that provided audio feedback when hit. A 27-inch monitor was placed behind the drums that played a custom-designed animation of four coconuts falling from a palm tree and hitting the ground (see Figure 1) synchronized with music chosen to have a specific music time signature (4/4) and which were instrumental versions of two Bob Marley songs. The actual rate that the coconuts dropped varied between 0.993 and 0.967 Hz because of the frame rate of the display.

Reflective markers were placed on the skin bilaterally on the dorsal midpoints of the wrists, second and fifth metacarpals, and fingernails of the thumb and index fingers, to track upper limb joint motion using 12 motion capture cameras (Vicon Motion Systems, Denver, CO, United States). Digital video was captured at the same time. A kinematic model was constructed in Visual 3D software (C-Motion, Inc., Germantown, MD, United States) to track hand path.

A 64-channel, wireless EEG system (Brain Products, Morrisville, NC, United States) was used with the cap positioned according to the 10–20 international system. EEG data were referenced to FCz and collected at 1000 Hz. Two minutes of seated rest were collected as baseline.

#### 2.2.3 Data acquisition

During the experiment, participants were instructed to rest the designated hand(s) on the table in front of the drums, watch the video, hit the drums when the coconuts hit the ground, and then return the hand to rest. This process was repeated eight times, which constituted a single trial for each of four drumming conditions: (1) with the dominant hand (dominant); (2) with the non-dominant hand (non-dominant); (3) with both hands together (synchronous); (4) with alternating hands (asynchronous) for each of the two songs. The intertrial interval was randomly set to a value between 4 and 5 s. For each condition, 50 trials were completed, with the order of conditions randomized.

#### 2.2.4 Video and motion data analysis

Video analysis provided information on whether the task was completed successfully, with success being defined as four hits with the designated hand(s) per trial. For trials that contained five or more hits, only the first four were retained. For trials with three or fewer hits, the hits were aligned with the closest stimulus time. A kinematic model was constructed in Visual 3D (C-Motion Inc., Germantown, MD) software. The kinematic data were then extracted to MATLAB (MathWorks, Natick, MA) for further analysis of the timings of the hand-drum contacts in relation to the coconut drops (i.e., timing) as well as how successfully the slightly less than 1 Hz drumbeat was maintained (i.e., cadence). Timing of the hit was calculated as the absolute value of the difference between the time at which the coconut hit the ground in the animation and the time at which the hit occurred, while cadence was calculated as the mean difference between the first and second hits, the second and third hits, and the third and fourth hits. Each participant was given a timing and cadence score for each condition.

#### 2.2.5 EEG data analysis

EEG data were processed in MATLAB with the EEGLAB toolbox as described in previous publications, Hinchberger et al. (2023) and Phillips et al. (2023). Line noise at 60 Hz and 120 Hz was removed using the Cleanline function. Channels were also removed if they demonstrated a flatline for longer than 5 s, insufficient correlation with neighboring channels (*r* < 0.7), or if noise contamination caused the channel to exceed a kurtosis of four standard deviations from the mean. If a channel was removed for one condition, it was removed for all conditions. Datasets were then merged and down sampled to 250 Hz. Artifact subspace reconstruction (ASR) was applied, which removed non-stereotypical artifacts from the dataset (Mullen et al., 2013). Using visual inspection, noisy time periods were also removed before Adaptive Mixture Independent Component Analysis (AMICA) was performed. These ICA matrices computed from ASR cleaned datasets were next applied to the same preprocessed individual datasets that did not have ASR applied. The EEGLAB DIPFIT algorithm was then used to compute dipoles for each independent component (IC) (Oostenveld and Oostendorp, 2002). Independent components (ICs) that displayed a topographical sparseness greater than 5 or a residual variance greater than 20% were excluded from further analysis (Bulea et al., 2015; Melnik et al., 2017). Non-cortical components were rejected using ICLabel and visual inspection of their topography and power spectrum (Pion-Tonachini et al., 2019). Time series task-related data from the retained cortical components were epoched from 1,000 ms before the video started to 4,000 ms after the first hit. The 2-min rest data were also epoched in 5000 ms increments to match the epoch length of the experimental data. Before clustering, dipoles for left-hand dominant participants were flipped for group-level alignment. *K-*means clustering was then performed. Principal Component Analysis (PCA) was used to determine the parameters for k-means clustering. Power spectra from 2 to 50 Hz, event-related potentials (ERPs), and inter-trial coherence (ITC) were assigned three PCA dimensions with a weight of 1 for clustering while dipole locations were assigned three PCA dimensions with a weight of 3 for clustering. ICs that were more than three standard deviations from any cluster centroid were relegated to an outlier cluster. ICs within clusters were separated by group (TD and CP). Additional non-cortical components were removed based on visual inspection of their dipole location.

In our previous studies where there were multiple ICs from the same person in a cluster, ERD values were averaged across ICs (Hinchberger et al., 2023). However, we wanted to devise a more objective and quantifiable method here with the goal to maximize the chances that each cortical cluster was representative of similar brain processes across individuals within it. The power spectral densities (PSDs) were computed across all trials for all ICs from a single participant that were part of the same cluster and were visually compared to the cluster’s mean PSD to identify the IC with the highest similarity to the mean across the frequencies of interest. If the case arose where more than one IC was similar to the cluster mean, we computed a score for each IC that summed together the z-scores of: ICLabel, percent residual variance (RV), and topographical sparseness values as shown below. The mean and standard deviation values for the ICLabel, RV, and topographical sparseness that were used to compute the z-scores came from the other ICs that were a part of that same cluster. The IC with the lowest score was retained in the analyses.


$$\begin{array}{l} \text{Score}=Z\;\text{Score of}\;(100-\text{ICLabel})+Z\;\text{Score of}\;\mathrm{RV}\\ +Z\;\text{Score of Topographical Sparseness} \end{array}$$

For the rest and drumming trials, the power in each IC was calculated by multiplying the time frequency data by its complex conjugate and converting to decibels. We created event-related spectral perturbations (ERSPs) relative to rest by dividing the power in the experimental condition by the mean power per frequency for the rest condition. For each IC and condition, we calculated the mean ERSP across all epochs. For each cluster and participant, ERD values were extracted from the alpha (6–12 Hz), beta (13–30 Hz), and gamma (31–50 Hz) frequency bands for the time period starting with the first and ending with the last hit, for each condition.

### 2.3 Statistical analysis

BBT, motion capture, and EEG data were analyzed using IBM SPSS Statistical Software v30. Generalized Linear Models (GLMs) were fit to compare data between conditions and groups with a significance threshold at *p* < 0.05. *Post hoc* tests were performed when indicated using independent or paired-samples *t*-tests.

Pearson correlations were performed as an exploratory analysis to identify significant relationships between BBT and EEG outcomes with a threshold of *p* < 0.05 with no correction for multiple comparisons.

## 3 Results

### 3.1 Motor performance results

**Figure 2:**
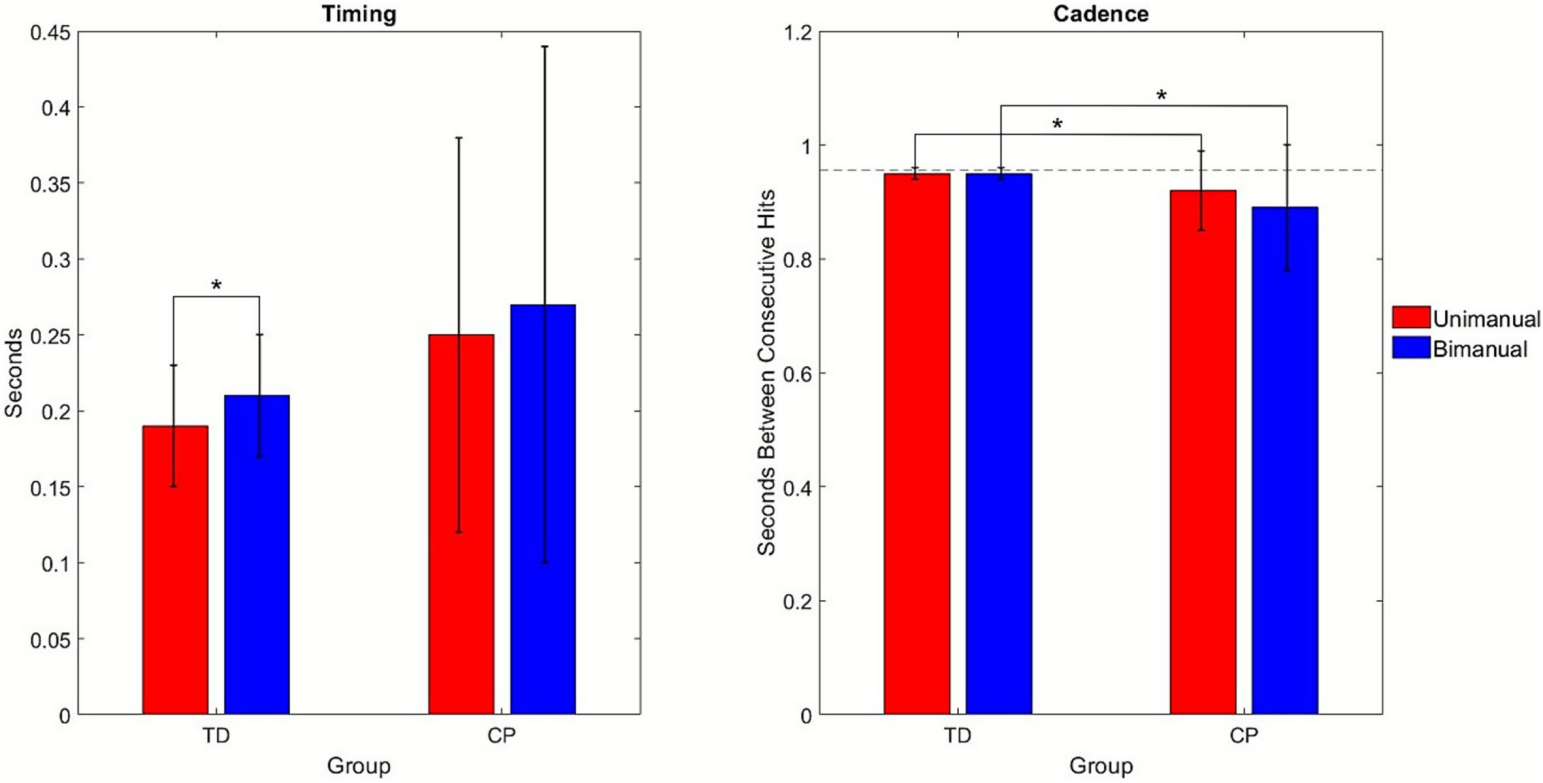
Bar graphs comparing the results for the mean timing (left) and cadence (right) by condition, unimanual (red) and bimanual (blue), and group, TD and CP. *, *p* < 0.05.

The percent of participants who had exactly four hits in a given trial, and the timing and cadence of those hits were quantified for each trial using the motion capture data and averaged across groups and bimanual (synchronous and asynchronous) and unimanual (dominant and non-dominant) conditions (Figure 2). A GLM was initially performed across all four conditions for these three measures, but this failed to demonstrate a main effect for condition or interaction between condition and group, although some comparisons showed a group main effect. Since our primary goal of the study was to compare unimanual and bimanual performance, we decided to combine the unimanual conditions when comparing to the bimanual conditions because both hands are involved in the bimanual tasks. We also decided to combine the bimanual conditions to see if this would enhance the effect observed in the mean differences across groups. These results are summarized in Table 2.

**Table 2:** Summary of general linear model *p*-values for condition (unimanual and bimanual), condition by group, and group effects for percent with four hits, timing and cadence.

Test	Condition	Condition by group	Group
Percent with four hits	0.86	0.89	0.04*
Timing	0.08	0.64	0.27
Cadence	0.24	0.49	0.07

*, *p* < 0.05.

The number of trials with fewer or more than four hits was also recorded by group. The group with TD had 13 incorrect trials, 8 with too few and 5 with too many hits, for 99.2% of trials having exactly four hits. One person in that group had eight of the incorrect trials, four trials with too few, and four with too many hits. The group with CP in contrast had 117 incorrect hits, 85 too few and 32 too many for 94.2% of trials having exactly 4 hits. Two participants with CP accounted for most of the incorrect hits with one having 24 and a second having 37 trials with too few hits and the latter participant also having 19 trials with too many hits. The mean correct percentages across the dominant, non-dominant, synchronous and asynchronous conditions were as follows: 98.4, 98.4, 98.3, 99.4 for the group with TD and 90.5, 89.7, 87.3, 93.1 for the group with CP, respectively. The GLM for percent correct showed only a significant main effect for group.

Supplementary Table 1 provides the mean data by group for the unimanual and bimanual conditions along with the mean differences between groups, confidence intervals and Cohen’s d effect sizes. Participants, regardless of group, had more accurate timing in the unimanual task (0.22 ± 0.10 s) compared to bimanual (0.24 ± 0.13 s). *Post hoc* analyses revealed that the conditions differed in TD (*p* = 0.02) but not CP even though the mean changes were similar. Cadence tended to be slower (*p* = 0.08 for group main effect) in CP for both conditions compared to TD. The group with CP also demonstrated greater variability for both timing and cadence in both conditions.

The Box and Block Test (BBT) showed significant differences across groups for both the dominant (58.08 ± 7.86 and 42.71 ± 11.91 for TD and CP, respectively; *p* < 0.001) and non-dominant (56.62 ± 6.89 and 30.36 ± 17.67 for TD and CP, respectively; *p* < 0.001) hand. Only the group with CP had a significant difference between dominant and non-dominant hands (42.71 and 30.4, respectively; *p* = 0.01).

### 3.2 EEG results

Including all data across conditions and participants, nine total brain clusters were identified. Table 3 provides the cluster location, based on the centroid location in Talairach coordinates, topographical maps, and the number of participants and group percentage for each cluster. A pattern was observed where the dominant frontal, premotor and primary motor hemisphere clusters had a greater percentage of CP subjects represented as did the non-dominant sensorimotor cluster. The remaining non-dominant clusters and the dominant posterior parietal cluster had a greater percentage of TD representation.

**Table 3:** Overview of EEG-computed clusters of cortical activity identified during the drumming tasks.

Cluster	Scalp topography	Centroid (MNI x,y,z)	Brodmann areas^1^	Number of participants		Percent of participants (%)	
				TD	CP	TD	CP
Dominant primary motor	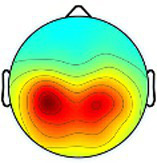	−36,−21,55	3,4	8	10	61.5%	76.9%
Non-dominant primary motor	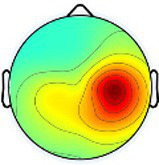	42,-15,48	2,3,4	9	6	69.2%	46.2%
Dominant premotor	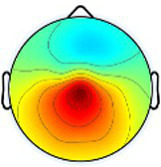	-2,2,64	6	10	11	76.9%	84.6%
Non-dominant prefrontal	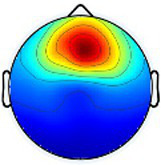	18,39,31	6,9,46	10	8	76.9%	61.5%
Dominant frontal	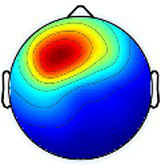	−25,35,31	8,9,46	5	8	38.5%	61.5%
Non-dominant sensorimotor	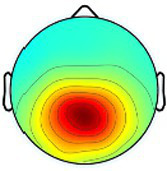	9,-39,59	5,7	7	8	53.8%	61.5%
Non-dominant occipital	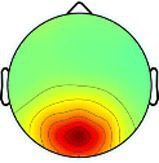	5,-79,35	7,18,31	11	6	84.6%	46.2%
Dominant posterior parietal	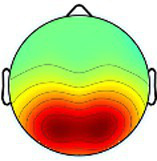	−28,-53,35	7,39	12	10	92.3%	76.9%
Non-dominant posterior parietal	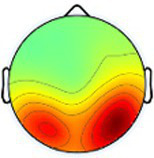	40,-54,23	7,22,39,40	9	8	69.2%	61.5%

1 Areas within +/− 5 mm cube of cluster centroid in the Talairach Atlas.

**Figure 3:**
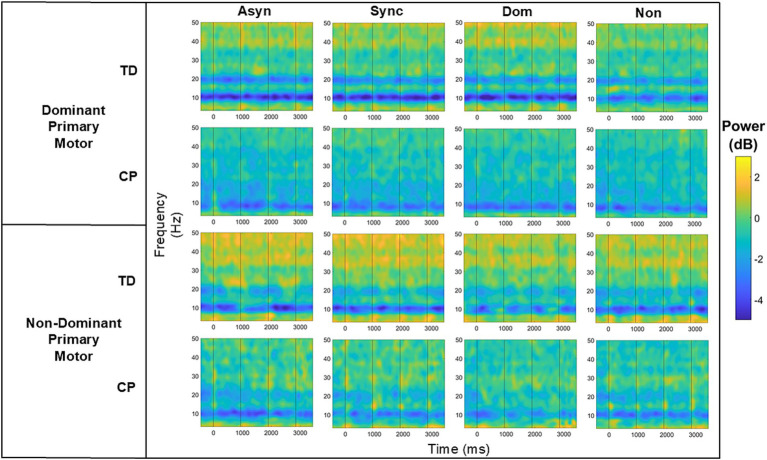
Event-related spectral perturbations (ERSPs) of the dominant and non-dominant primary motor clusters for each group and condition with a universal power bar ranging from −5.0 dB to 3.0 dB to maintain visual consistency, with the vertical lines signifying the median time when the first, second, third, and fourth hits occurred for all participants in all trials. Each ERSP for each cortical component was time-warped to those median values for the task-related data by condition so that data across participants within a specific group (TD, typically developing; CP, cerebral palsy) were aligned. Asyn, asynchronous; Sync, synchronous; Dom, dominant; Non, non-dominant.

Event-related spectral perturbation (ERSP) plots for the dominant primary motor cluster revealed the most consistent and highest magnitude ERD in the alpha band across conditions within the group with TD, with consistent, although less, ERD in the beta band for the same conditions (Figure 3). Consistent but relatively less ERD was observed in these same bands for the non-dominant motor cluster in TD across all tasks. Collectively, beta ERD was present in all conditions but more tightly distributed around the peak frequency in the group with TD compared to CP. Like TD, alpha band ERD was more consistently present across the four tasks and both hemispheres in the group with CP compared to beta ERD. Finally, low gamma band ERS was generally more prevalent across tasks in TD compared to CP, especially in the non-dominant cluster.

**Figure 4:**
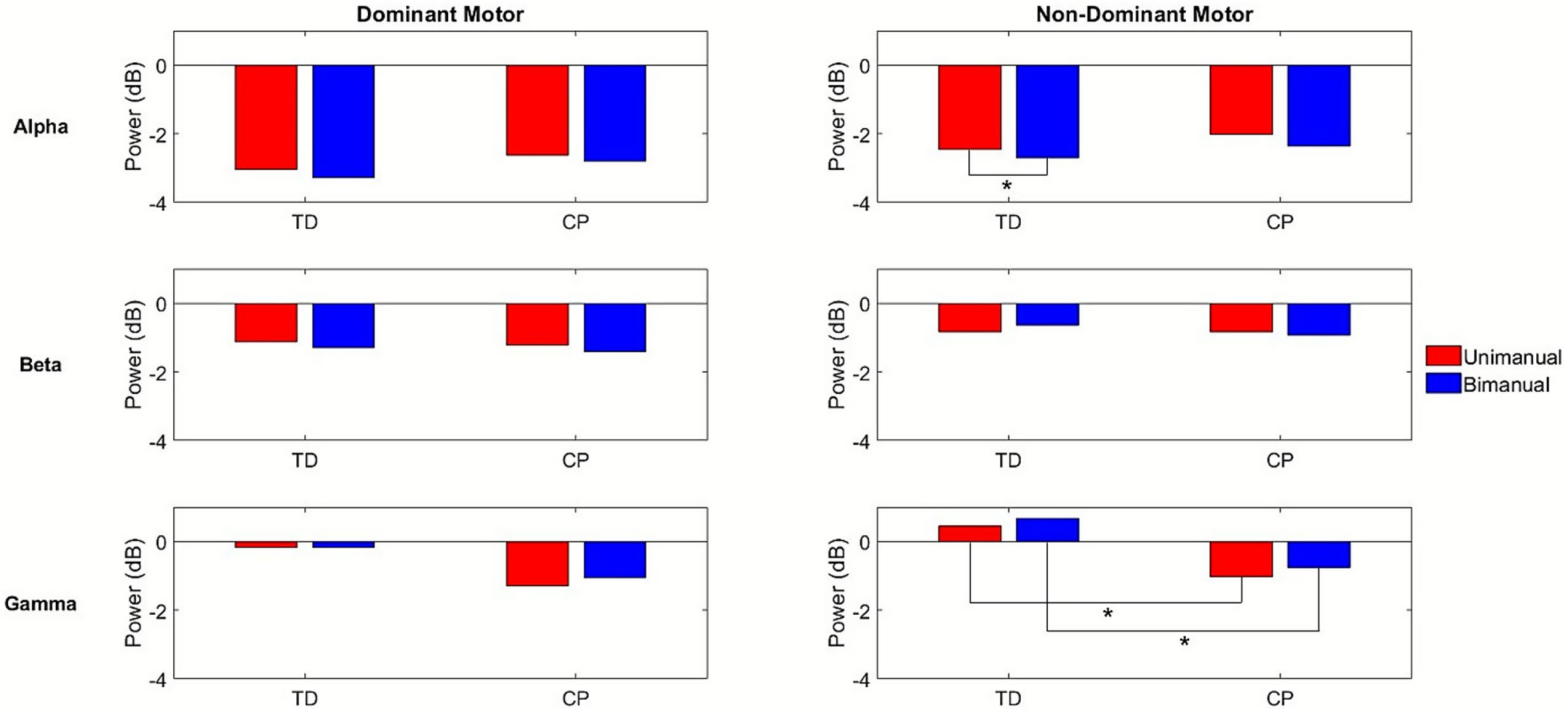
Bar graphs comparing the results for the mean alpha (top row), beta (middle row), and gamma (bottom row) event-related desynchronization (ERD) or event-related synchronization (ERS) in the dominant motor (left column) and non-dominant motor (right column) clusters for conditions unimanual (red) and bimanual (blue) and group. *, *p* < 0.05.

To compare ERSPs across conditions and groups, we first fit a GLM with both the dominant and non-dominant primary motor clusters, to evaluate whether there was an effect for hemisphere, but no significant main effect or interaction was found. We also initially conducted the GLMs with all four conditions but found no main effects for condition or condition by group interactions but noted again that the trends were similar in the two unimanual conditions and the two bimanual conditions, so we combined these to increase the magnitude of the effect to address the primary goal of comparing bimanual and unimanual tasks and repeated the analyses. These new values were calculated by averaging the ERD or ERS values for the dominant and non-dominant unimanual conditions and the ERD or ERS values for the asynchronous and synchronous bimanual conditions, seen in Figure 4 for the dominant and non-dominant motor clusters as an example.

Table 4 summarizes the GLM analyses for all clusters broken down by frequency band and *p*-values for group, condition (unimanual or bimanual), and condition by group effects with the significant p-values in bold.

**Table 4:** Summary of general linear model *p*-values for condition, condition by group, and group effects for all the clusters for the EEG data at each frequency.

Cluster	Number of TD	Number of CP	Frequency	Condition	Condition by group	Group
Dominant primary motor			Alpha	0.13	0.86	0.83
	8	10	Beta	0.08	0.85	0.60
			Gamma	0.38	0.58	0.17
Non-dominant primary motor			Alpha	0.03*	0.67	0.72
	9	6	Beta	0.61	0.16	0.76
			Gamma	0.22	0.88	0.01*
Dominant premotor			Alpha	0.48	0.45	0.90
	10	11	Beta	0.79	0.51	0.15
			Gamma	0.02*	0.56	0.07
Non-dominant prefrontal			Alpha	0.45	0.10	0.87
	10	8	Beta	0.37	0.94	0.25
			Gamma	0.68	0.16	0.13
Dominant frontal			Alpha	0.39	0.34	0.93
	5	8	Beta	0.84	0.77	0.08
			Gamma	0.81	0.87	0.17
Non-dominant sensorimotor			Alpha	0.01*	0.17	0.37
	7	8	Beta	0.04*	0.91	0.37
			Gamma	0.91	0.39	0.09
Non-dominant occipital			Alpha	0.49	0.01*	0.12
	11	6	Beta	0.98	0.47	0.01*
			Gamma	0.10	0.70	0.01*
Dominant posterior parietal			Alpha	0.39	0.91	0.69
	12	10	Beta	0.97	0.30	0.13
			Gamma	0.39	0.55	0.06
Non-dominant posterior parietal			Alpha	0.64	0.14	0.47
	9	8	Beta	0.66	0.88	0.29
			Gamma	0.23	0.93	0.01*

*, *p* < 0.05.

#### 3.2.1 Alpha band

There was a significant main effect for condition in the alpha band of the non-dominant primary motor cluster (*p* = 0.03), with *post hoc* testing indicating that, in the TD group, the bimanual condition had significantly greater ERD magnitude (−2.70 ± 2.27 dB) compared to the unimanual condition (−2.47 ± 2.40 dB). A similar trend was observed in the group with CP, where the bimanual condition had greater ERD magnitude (−2.35 ± 1.74 dB) compared to the unimanual condition (−2.02 ± 1.40 dB). A significant main effect for condition was also seen in the non-dominant sensorimotor cluster (*p* = 0.01), where the bimanual condition displayed a significantly greater ERD magnitude, only in the TD group, (−1.87 ± 2.12 dB) compared to the unimanual condition (−0.85 ± 1.96 dB). Again, there was a similar trend where the bimanual condition had a greater magnitude of ERD compared to the unimanual condition in the group with CP. A significant condition by group effect in the non-dominant occipital cluster (*p* = 0.01) was found with the TD group displaying a greater magnitude ERD for the bimanual condition (−0.79 ± 0.94 dB) compared to unimanual (−0.40 ± 0.93 dB) while the group with CP displayed a lower magnitude ERD for the bimanual condition (−1.50 ± 1.73 dB) compared to unimanual (−1.72 ± 1.67 dB).

#### 3.2.2 Beta band

There was a significant main effect for condition in the beta band of the non-dominant sensorimotor cluster (*p* = 0.04) for the TD group, where the bimanual condition displayed a significantly greater ERD magnitude (−0.62 ± 0.81 dB) compared to unimanual (−0.38 ± 0.74 dB). Similarly, the bimanual condition had greater ERD magnitude than the unimanual condition in the group with CP. There was also a significant group effect in the non-dominant occipital cluster (*p* = 0.01), in which the group with CP displayed ERD while the group with TD displayed ERS for both the bimanual and unimanual conditions.

#### 3.2.3 Gamma band

There was a significant main effect for group in the non-dominant primary motor cluster (*p* = 0.01) where the group with CP displayed ERD while the group with TD displayed ERS for both the bimanual and unimanual conditions. There was another significant group effect for the non-dominant occipital cluster (*p* = 0.01) and the non-dominant posterior parietal cluster (*p* = 0.01) where again the group with CP displayed ERD while the group with TD displayed ERS for both the bimanual and unimanual conditions.

There was a significant main effect for condition in the dominant premotor cluster (*p* = 0.02) where the TD group displayed greater ERS in the bimanual (0.49 ± 0.55 dB) compared to the unimanual (0.29 ± 0.48 dB) condition.

### 3.3 Correlations

EEG outcome measures (nine clusters, four conditions, three frequency bands) were correlated with the Box and Block test within groups (see Table 5).

**Table 5:** Pearson correlation coefficients (r) for all significant results (*p* < 0.05) between the EEG values by group, cluster, frequency band, and the Box and Block Test.

Group	Cluster	Frequency	Condition	Dom BBT	Non BBT
TD	Dominant premotor	Gamma	Dom	0.73	
TD	Non-dominant prefrontal	Beta	Dom	0.79	
CP	Dominant primary motor	Alpha	Dom	−0.67	
		Beta	Dom	−0.70	
CP	Non-dominant primary motor	Alpha	Dom	−0.93	
CP	Dominant premotor	Beta	Dom	−0.65	
CP	Non-dominant prefrontal				
		Beta	Dom	−0.91	
			Non		−0.81
CP	Dominant posterior parietal		Dom	−0.68	
			Non		−0.69
		Beta	Non		−0.74

TD, typical development; CP, cerebral palsy; Dom, dominant; Non, non-dominant; BBT, Box and Block Test.

For the group with TD, there was a positive correlation between EEG activity and the BBT (lower (or lower negative) ERD in the alpha and beta band and greater ERS in the gamma band were related to better BBT scores) while the group with CP exhibited a negative correlation (higher alpha and beta ERD (or higher negative) values were related to better BBT scores) in all cases.

## 4 Discussion

The goal of this study was to examine differences in brain activity and task performance during bimanual and unimanual drumming in children with bilateral CP and TD. This study is unique in that it combined quantitative measures of motor performance with high density EEG to study bimanual coordination in children with bilateral CP, rather than those with unilateral CP. CP is a heterogeneous condition with a wide range of types and severity of the brain injuries across and within subgroups. How the immature brain reorganizes in response to a unilateral versus bilateral brain injury is fundamentally different (Kolb et al., 2013), thus we anticipated that those with bilateral CP would show different patterns of brain activation, similar to those found in our earlier fNIRS study where we compared data during an upper and lower limb task across these two CP types and to TD (Sukal-Moulton et al., 2020). However, even when the type of brain injury is similar (e.g., neonatal stroke or periventricular leukomalacia) the outcome can still vary widely across individuals (Weinstein et al., 2018; Damiano et al., 2022) for reasons not yet fully understood. Therefore, although we are reporting on group differences here, ultimately we propose that imaging data from an individual could be utilized to guide more personalized rehabilitation care.

This study is also different from EEG studies in bilateral CP by other investigators because cortical sources were clustered based on anatomical location and activation onset, which is particularly useful in evaluating children with brain injures (Hinchberger et al., 2023). Since the brain injury may distort brain anatomy compared to those without brain injury, this process helps to increase confidence that we are comparing similar regionally based brain processes.

We hypothesized that motor performance and associated brain activation in participants with CP would show greater differences from those with TD during bimanual compared to unimanual tasks based on increased complexity of using two versus one hand and differences between groups may become more apparent when the task is more challenging. With respect to motor performance, there was a trend for poorer timing of the hit with respect to the visual cue during bimanual compared to unimanual tasks with groups combined but it was not significantly different for condition or between groups. Cadence, or maintaining the beat, tended to be worse in the group with CP compared to TD in both conditions with the mean difference slightly but not significantly greater for the bimanual condition.

Similarly, the EEG results for the bimanual tasks did not demonstrate differentially worse effects by group on brain activation than unimanual ones during the drumming tasks, with one exception: the group with bilateral CP showed less activation in the bimanual compared to unimanual conditions in the alpha band of the non-dominant occipital region, which was an unusual finding. One possible explanation for this may be the higher prevalence of central vision impairment (CVI) which is 10–70 times more common in children with CP compared to the general pediatric population, with almost 66% of children with CP presenting with CVI (Kozeis and Jain, 2025). CVI affects the visual processing pathways without damage to the eyes themselves (Fazzi et al., 2012) which may have had a particular effect on disrupting pathways that control eye hand coordination in bimanual tasks, leading to fewer pathways being activated. Our results are like those from other EEG studies on the occipital region in patients with CVI. Kriegseis et al. (2006) found a decrease in alpha activity in the parieto-occipital region in participants with CVI during a somatosensory perception task and a mental imagery task. A more recent study by Federici et al. (2023) found an increase in the alpha band activity in the occipital region during the more complex visual tasks for their control group, but not in the group with CVI.

Of note, all other significant between-group differences where the group with CP demonstrated significantly greater task-related activity in the beta band and less in the gamma frequency band than TD were in non-dominant brain regions, regardless of condition. Furthermore, there were trends in both groups towards greater brain activation in the alpha and beta frequency bands with more activity in the gamma band for bimanual compared to unimanual conditions.

We had anticipated that the asynchronous condition would be particularly difficult in those with CP; however, when we compared all four conditions, results were very similar for the synchronous and asynchronous conditions, both of which are symmetric bimanual tasks (Woytowicz et al., 2016). Our results here were in contrast to an earlier fNIRS study by our group (de Campos et al., 2020) demonstrating significant group differences between those with unilateral CP and TD for a task where one hand poured into a cup held by the other hand. Asymmetric tasks such as that one where two hands perform two different tasks at the same time are far more challenging, and perhaps more effective in differentiating groups, and should be considered for future bimanual studies in bilateral CP. Another reason for the lack of a difference between the two bimanual conditions in either CP or TD may be that any timing differences between hands during a unimanual task would similarly affect both bimanual tasks regardless of the phase.

Children with bilateral CP have shown significant differences on both sides as well as asymmetry across sides in motor performance and brain activity during unimanual fine motor tasks compared to TD, although these tend to be less pronounced than in unilateral CP (Hinchberger et al., 2023; Phillips et al., 2023). The present study, however, saw increased brain activation in the group with CP only in the non-dominant occipital region in the beta frequency band while Hinchberger et al. (2023) and Phillips et al. (2023) saw increased brain activation in both primary motor hemispheres. One difference here was that the task was a gross motor one where participants used the whole hand to pat the drum versus a fine motor one involving reaching to grasp or transfer a cube. Since children with CP tend to have greater distal impairments, the drumming task may not have been as challenging, thus limiting significant differences.

The exploratory correlation analyses revealed divergent results across groups. Better BBT scores in TD were related to less brain activity in alpha and beta, which indicates that higher performers tend to have more efficient neural activation, similar to the finding in Puttemans et al. (2005) which showed less activation with increased learning of a task. In contrast in CP, better BBT scores were related to higher brain activity as we have seen previously in this cohort suggesting that increased brain “effort” may instead be a compensatory mechanism for their motor limitations or the result of differences in brain organization during development (Phillips et al., 2023). The direction of the relationship between motor performance and the magnitude of brain activation has been shown to vary with age, the specific task, motor skill level, frequency band, and brain region, among other factors in addition to the presence or absence of a brain injury (Phillips et al., 2023).

Children with CP had less brain activation than TD in the gamma band that may be related to poorer visual–spatial attention on average in that group. The gamma band group differences found here were not seen in our previous reaching study on children with bilateral CP (Hinchberger et al., 2023), which may be related to the addition of the bimanual tasks and to the visuomotor aspect which both increase the degree of sensorimotor integration needed. Greater synchronization of different brain regions and processes leads to better motor control, and the gamma band represents a core mechanism to coordinate these (Ulloa, 2022).

### Limitations

This study contained a relatively small sample of 13 children with bilateral CP and 13 children with TD, which is modest; nevertheless, multiple significant EEG differences, behavioral differences, and correlations uncorrected for multiple comparisons were uncovered.

Another limitation was that only the participants with bilateral CP who were able to complete the tasks with both hands were included in this study. This excluded participants with greater motor impairment, so group differences may likely have been larger in a more representative sample of children with bilateral CP.

Children with brain injuries may have significant anatomical differences that make it challenging to compare data in specific brain regions to others with or without brain injuries. For example, many children with bilateral CP have enlarged ventricles as a result of intraventricular hemmorhages that shift the sensorimotor cortical areas laterally. EEG due to volume conduction is known to have lower spatial resolution than other imaging techniques; however, independent component analysis (ICA) improves localization of brain activity even in the presence of anatomical differences. ICA accomplishes this by separating the recorded signals from the scalp into distinct, statistically independent and more compact and functionally related brain processes (or external processes like eye blinks or neck muscle activity). This along with K-means clustering of brain-related ICs increases confidence in the comparison of regional brain activity across participants. Another possible limitation was the selection of a single IC from a cluster for each participant even when multiple ICs were found. There is no consensus on how to best handle this situation. In one of our previous studies (Hinchberger et al., 2023), if a cluster had multiple ICs from a participant, the ERD values for all ICs were averaged. The present study instead aimed to identify the IC that best represented the cluster. We developed a score that rated the relative similarity of each IC to the activity of the entire cluster by summing a normalized value of three objective criteria. The IC with the lowest score was retained and the other ICs were removed from the cluster. We believe that this novel method can be used to determine the cortical process most closely aligned with others in the cluster identified by the k-means algorithm, but this issue warrants more discussion in the field.

## 5 Conclusion

This is the first EEG study to our knowledge examining brain activation during upper limb bimanual coordination tasks exclusively in children with bilateral CP in comparison with TD. As found in our previous studies of unimanual tasks in bilateral CP, bimanual tasks also showed worse motor performance and higher brain activation in the alpha and beta band in CP compared to TD. While we expected that bimanual tasks would more clearly differentiate children with CP from those with TD, this was not the case even though the bimanual conditions were generally more challenging for both groups. Two novel findings included: (1) greater group differences in the gamma band likely related to the specific task, which required participants to respond to a visual cue to execute a movement, thus entailing more sensorimotor integration; and (2) disparate findings in CP for the non-dominant occipital region, which we speculated were related to high prevalence of CVI in those with CP. Finally, this study reinforces previous conclusions from our other studies in bilateral CP of higher brain activation being associated with greater motor deficits (Hinchberger et al., 2023; Lee et al., 2025) and showing that children with bilateral CP have significant unimanual and also bimanual impairments that deserve greater clinical recognition and remediation.

## Data Availability

The raw data supporting the conclusions of this article will be made available by the authors, without undue reservation.

## Author disclaimer

The contributions of the NIH author(s) are considered Works of the United States Government. The findings and conclusions presented in this paper are those of the author(s) and do not necessarily reflect the views of the NIH or the U.S. Department of Health and Human Services.
